# Integrated community case management and community-based health planning and services: a cross sectional study on the effectiveness of the national implementation for the treatment of malaria, diarrhoea and pneumonia

**DOI:** 10.1186/s12936-016-1380-9

**Published:** 2016-07-02

**Authors:** Blanca Escribano Ferrer, Jayne Webster, Jane Bruce, Solomon A. Narh- Bana, Clement T. Narh, Naa-KorKor Allotey, Roland Glover, Constance Bart-Plange, Isabella Sagoe-Moses, Keziah Malm, Margaret Gyapong

**Affiliations:** Disease Control Department, London School of Hygiene and Tropical Medicine, London, UK; Dodowa Health Research Center, Ghana Health Service, Dodowa, Ghana; School of Public Health, University of Health and Allied Sciences, Hohoe, Volta Region Ghana; National Malaria Control Programme, Ghana Health Service, Accra, Ghana; Reproductive and Child Health Department, Ghana Health Service, Accra, Ghana

**Keywords:** Home-based care, Community-based care, Integrated community case management (iCCM), Integrated management of childhood illness (IMCI), Malaria, Diarrhoea, Pneumonia, Children under-five

## Abstract

**Background:**

Ghana has developed two main community-based strategies that aim to increase access to quality treatment for malaria, diarrhoea and pneumonia: the Home-based Care (HBC) and the Community-based Health Planning and Services (CHPS). The objective was to assess the effectiveness of HBC and CHPS on utilization, appropriate treatment given and users’ satisfaction for the treatment of malaria, diarrhoea and pneumonia.

**Methods:**

A household survey was conducted 2 and 8 years after implementation of HBC in the Volta and Northern Regions of Ghana, respectively. The study population was carers of children under-five who had fever, diarrhoea and/or cough in the last 2 weeks prior to the interview. HBC and CHPS utilization were assessed based on treatment-seeking behaviour when the child was sick. Appropriate treatment was based on adherence to national guidelines and satisfaction was based on the perceptions of the carers after the treatment-seeking visit.

**Results:**

HBC utilization was 17.3 and 1.0 % in the Volta and Northern Regions respectively, while CHPS utilization in the same regions was 11.8 and 31.3 %, with large variation among districts. Regarding appropriate treatment of uncomplicated malaria, 36.7 % (n = 17) and 19.4 % (n = 1) of malaria cases were treated with ACT under the HBC in the Volta and Northern Regions respectively, and 14.7 % (n = 7) and 7.4 % (n = 26) under the CHPS in the Volta and Northern Regions. Regarding diarrhoea, 7.6 % (n = 4) of the children diagnosed with diarrhoea received oral rehydration salts (ORS) or were referred under the HBC in the Volta Region and 22.1 % (n = 6) and 5.6 % (n = 8) under the CHPS in the Volta and Northern Regions. Regarding suspected pneumonia, CHPS in the Northern Region gave the most appropriate treatment with 33.0 % (n = 4) of suspected cases receiving amoxicillin. Users of CHPS in the Volta Region were the most satisfied (97.7 % were satisfied or very satisfied) when compared with those of the HBC and of the Northern Region.

**Conclusions:**

HBC showed greater utilization by children under-five years of age in the Volta Region while CHPS was more utilized in the Northern Region. Utilization of HBC contributed to prompt treatment of fever in the Volta Region. Appropriate treatment for the three diseases was low in the HBC and CHPS, in both regions. Users were generally satisfied with the CHPS and HBC services.

**Electronic supplementary material:**

The online version of this article (doi:10.1186/s12936-016-1380-9) contains supplementary material, which is available to authorized users.

## Background

During the past 30 years, the under-five mortality rate has declined in Ghana from 145/1000 live births in 1998 to 60/1000 live births in 2014 with an infant mortality rate of 41/1000 and a neonatal mortality rate of 29/1000 live births. These mortalities are higher in the north of the country and in the rural areas. Despite this decline in under-five year mortality, the Millennium Development target of 40/1000 was not reached [1]. The main causes of under-five mortality are neonatal related causes (38 %), malaria (20 %), pneumonia (11 %) and diarrhoea (8 %) [2]. In 2012, the Child Survival Call to Action set “A Promise Renewed” with the target of decreasing under-five mortality rates to 20 or fewer deaths per 1000 live births by 2035 in all countries [2].

Access to anti-malarials within 24 h of the onset of malaria symptoms is vital to prevent progression to severe malaria or death. The Roll Back Malaria partnership recommends that 100 % of those suffering from malaria should have prompt access to affordable and appropriate treatment within 24 h of onset of symptoms [3, 4].

There are three key strategies that seek to improve physical access to quality treatment which are: extension and quality improvement of formal health care systems, improvement in the informal private sector (mainly drug shops), and the home-based care (HBC) of fevers [5]. The World Health Organization and the Roll Back Malaria partnership states that in settings with limited access to health facilities, diagnosis and treatment should be provided at community level through community case management of malaria, recommending the introduction of rapid diagnostic test (RDT) and rectal artesunate for referral, when possible [4, 6, 7]. Malaria HBC has been shown to be effective and cost effective especially in areas with high malaria transmission, and in areas with medium transmission and low coverage of health facilities [8–13]. Integrated HBC or integrated community case management (iCCM) does not reduce the quality of malaria case management if adequate training is provided and supervision is maintained [14]. Issues related to implementation (e.g., availability of CBAs, availability of drugs or access to facilities), may decrease the expected impact of the strategy. The United Nations Children’s Fund (UNICEF) and the World Health Organization officially endorsed iCCM in 2012 [15].

Ghana has developed two main community-based interventions or delivery strategies that aim to reduce barriers to physical access to quality treatment: the HBC and the community-based health planning and services (CHPS).

The HBC strategy started on a pilot basis in Ghana in 1999 to treat suspected malaria cases [16]. The pilot programme initially used chloroquine, shifting to artemisinin-based combination therapy (ACT) in 2005 [17]. In 2009 and in the context of integrated management of childhood illness (IMCI), Ghana developed the *Home Management of Malaria, ARI and Diarrhoea in Ghana* [16] also called iCCM. HBC (or iCCM) was defined as prevention, early case detection and prompt and appropriate treatment of fevers, ARI and diarrhoea in the community.

The HBC strategy corresponds to the lowest level of health care delivery in Ghana and it is designed to be implemented within the health system, with community-based agents (CBA) reporting their activities to care providers at the CHPS compounds (when existing) or to the next health facility level. All CBAs in the three northern regions (Northern, Upper East and Upper West Regions) provide treatment for malaria, diarrhoea and suspected pneumonia cases based on clinical symptoms and with the support of ARI timers for measuring the respiratory rate to diagnose pneumonia cases, mainly with the financial support of UNICEF. Those in the rest of the country have received the same training as the three northern regions but provide only malaria treatment with the support of the Global Fund to fight AIDS, TB and malaria (GFATM), and are supposed to refer diarrhoea and suspected pneumonia cases for further management. Other projects implemented by non-governmental organizations support integrated HBC on a smaller scale in different regions of the country. The HBC guidelines state that the service provided should be free, although some regions (such as the Northern Region) decided that users should give a small amount of money to CBAs to avoid risking lack of continuity and commitment of the strategy as experienced in other countries [8, 18, 19]. No target was set for iCCM utilization as a proportion of other delivery points for treatment of sick children.

The CHPS strategy started in 1999 after a pilot phase conducted in 1994 [20] attempting to respond to the 1978 Alma Ata Conference and the ‘Health for All’ principle. A key component of the CHPS strategy is that traditional leaders of the community must accept the CHPS concept and commit themselves to supporting it. The CHPS strategy is based upon a basic facility known as a community health compound, where health care is provided by a resident community health nurse or community health officer who also does a 90 days cycle visiting the communities she/he serves at least once within that period. The services provided include immunizations, family planning, supervising delivery (if trained staff available), antenatal/postnatal care, treatment of common diseases such as malaria, diarrhoea and acute respiratory infections (ARI) and health education. These services are free for those having a valid national health insurance card. No target was set for CHPS utilization as a proportion of other delivery points for treatment of sick children. The target for CHPS coverage is that a geographical area of a 4 km radius and between 4500 and 5000 persons should be covered by a CHPS [21, 22].

After several years of national implementation, there is the need to know how effective HBC and CHPS are at delivering care for children with fever, diarrhoea or cough. There are several studies that looked at the HBC in Ghana. However, most of these studies focused in few districts, looked particularly at malaria HBC and were conducted in a more “controlled” context [23–27]. This study aims to assess the effectiveness of the national implementation of HBC and CHPS in terms of utilization of services, appropriate treatment given and users’ satisfaction in the current context, without additional supervision, in a larger area and considering the management of fever, diarrhoea and cough for children under-five years old.

## Methods

### Ethics

Ethical approval was obtained from the Ghana Health Service-Ethical review committee (ID NO; GHS-ERC: 04/09/13) and from the Ethics Committee of LSHTM (ethics ref: 6442). Administrative approval was obtained from the respective regions and districts. Carers of children gave written consent to be interviewed.

### Study site

The Volta and Northern Regions were purposively selected. The principal researcher wanted to include a region implementing iCCM and one malaria only HBC, to have a better picture of HBC in Ghana. Based on this first requirement, the National Malaria Control Programme (NMCP) suggested the Volta and Northern Regions. The Volta Region targeted only rural districts for the HBC implementation and implements mostly malaria HBC (with the exception of some communities supported by NGOs which implement integrated HBC), despite all districts received drugs for the management of diarrhoea and suspected pneumonia in 2013. The Northern Region implements iCCM due to availability of funds from UNICEF. Based on the monthly activities reported through the routine monitoring information (District Health Information System-DHIMS II), the NMCP had some concerns on the low performance of iCCM in Northern Region compared to the other two northern regions (Upper East and Upper West Regions), although the iCCM coordinator in the Northern Region believed this low performance was due to under reporting of activities. In contrast, the NMCP was satisfied with the malaria HBC implementation in the Volta Region. Selecting one “good” and “bad” performing region was believed to be a good strategy to contrast results with those of DIMS II and to see possible differences that could help identify enablers and barriers of the HBC implementation in Ghana. The CHPS strategy is uniform across regions of the country.

The Volta Region has a malaria prevalence of 17 %, diarrhoea prevalence of 7.6 % and suspected pneumonia prevalence of 2.1 % in children under-five (MICS 2011). The rural population corresponds to 66 % of the total population. Two rainfall patterns occur in the southern area of the Volta Region, one major season is in April/July with a peak in June and one minor season is in September/November with a peak in October. The north of Volta Region has one rainy season—May to October with a peak in August.

The Northern Region has a malaria prevalence of 48 %, diarrhoea prevalence of 21.4 % and suspected pneumonia prevalence of 6.3 % in children under-five (MICS 2011) [28]. The rural population corresponds to 70 % of the total population. In the north the rainy season begins in May and ends in October [29]. Climatically, religiously, linguistically, and culturally, the Northern Region differs greatly from the politically and economically dominating regions of southern Ghana, and it is similar to the two other regions in the north of Ghana (Upper East and Upper West).

### Study design and sampling procedures

This was an observational study post intervention without controls using a cross sectional household survey. The effectiveness of the implementation of appropriate treatment was assessed against national guidelines. The study population were carers of children under-five years of age, who had fever, cough and or diarrhoea in the last 2 weeks prior to the interview.

The sample size was estimated using the standard formula for estimation of a proportion and adjusting for clustering: [3.84p(1 − p)/e^2^] × DE [30]. A prevalence of 50 % of the population who are satisfied with the strategies was used to obtain a conservative sample size and ensure sufficiency for the estimation of utilization of the community services and several outcomes. A design effect of 1.5 [31] and a precision of 5 % were used. Adding 10 % for non-response, the sample size required in each region was 633, giving a total sample size of 1267 households with a child with fever, diarrhoea or cough in the 2 weeks preceding the survey.

A stratified three-stage cluster survey was conducted in each region. In order to have the sample representative of the whole region, whilst being logistically feasible, regions were divided into three areas. From each area, two districts and from each district, four clusters were selected using probability proportional to size. Then, from each cluster, 27 households were selected, making a total of 648 in each region. To select the districts (first stage) the list of districts implementing HBC (all districts implement the CHPS strategy) with its population was used. To select the clusters (second stage) the list of communities implementing HBC with its population was used. Households with children under-five that had fever, diarrhea or cough in the last 2 weeks prior to the interview were randomly selected in each cluster using a modified expanded programme on immunization sampling technique (third stage) [32]. To select households, a location near the centre of the community was first identified and a random direction was defined by spinning a pen. A random household along the chosen direction pointing outwards from the centre of the community to its boundary was chosen and checked for compliance with the inclusion and exclusion criteria. Whether the criteria were met or not, the next closest household was visited until the required number of households with a child with a fever, diarrhoea or cough in the 2 weeks preceding the survey were surveyed. Interviews were conducted with the carer of the sick child. In cases where there was more than one eligible child in a household, only one was selected randomly by ballot paper.

### Data collection

Data collection was done during the 5th to 16th April 2014 in the Volta Region and during the 23rd June to 3rd July 2014 in the Northern Region. Three teams of four field workers with one field supervisor were recruited in Dodowa township for the Volta Region data collection and in Tamale township for the Northern Region data collection. The recruitment followed a standard procedure which included an interview, previous experience as a field worker in DHRC and secondary education level. The training was done in Dodowa for the Volta Region team and in Tamale for the Northern Region team. The training was for a week and included 1-day pilot testing of the questionnaire. The same field supervisors and the trainers were used in both regions.

Data collection was done using a structured questionnaire, which included socio-demographic information of the care taker, care-seeking behaviour, experience with CBAs and other health providers, knowledge of the three diseases and household characteristics.

### Definitions

Appropriate provider refers to public or private medical facility, CHPS, CBAs or licensed chemical shop [28]. HBC is delivered by CBAs. Utilization of HBC or CHPS is defined as carers taking their child under-five to a CBA or a CHPS, respectively, when the child has symptoms of fever, cough or diarrhoea.

Flexibility of time of a CBA or of a health facility to attend a child refers to “open hours”, meaning the moments during the day that a child can be seen by a provider.

User satisfaction refers to carers experience with the service received after the treatment-seeking visit. Definitions specific to case management of malaria, pneumonia and diarrhoea, and their differentials by HBC and CHPS used in the study are presented in Table 1.

**Table 1:** Study definitions

Definitions	HBC [16]	CHPS [53]
Malaria	All fever cases when no laboratory tests are available	All fever cases when no laboratory tests are available or when malaria test was positive
General danger signs	Vomiting, convulsions, unconscious or not breastfeeding	Vomiting, convulsions, unconscious or not breastfeeding
Severe malaria signs	Little or no urine, dark coloured urine, marked jaundice or abnormal bleeding	Little or no urine, dark coloured urine, marked jaundice or abnormal bleeding
Appropriate treatment of malaria	Children aged 6 months to 5 years diagnosed with malaria receiving 3 days of ACT If more than 7 days with fever, general danger signs or severe malaria signs, child must be referred with rectal artesunate	Children aged 2 months to 5 years diagnosed with malaria receiving 3 days of ACT If more than 7 days with fever, general danger signs or severe malaria signs, child must be referred with IM quinine, IM or EV or rectal artesunate plus an IM dose of chloramphenicol
Prompt treatment of malaria	Malaria cases that received an antimalarial drug in within the first 24 h of the onset of symptoms	Malaria cases that received an antimalarial drug in within the first 24 h of the onset of symptoms
Diarrhoea	Three or more loose or watery stools in a 24-h period	Three or more loose or watery stools in a 24-h period
Appropriate treatment of diarrhoea	Children older than 6 months with diarrhoea of less than 7 days that receive ORS and zinc for 14 days If the child is less than 6 months, had diarrhoea for 7 days or more, blood in stools or is dehydrated, he/she should be referred with ORS	Children with diarrhoea of less than 14 days receiving ORS and zinc for 14 days If diarrhoea for 14 days or more, blood in stools or is severely dehydrated, he/she should be referred to hospital with ORS
ARI or suspected pneumonia	Cough with fast or difficult breathing^a^	Cough with fast or difficult breathing^b^
Severe pneumonia	Noisy breathing or chest in-drawing	Noisy breathing or chest in-drawing
Appropriate treatment for suspected pneumonia	Children older than 6 months with cough and fast or difficult breathing of less than 7 days receiving amoxicillin for 5 days If the child is less than 6 months or had symptoms for 7 days or more, he/she should be referred If there are signs of severe pneumonia, he/she should be referred with amoxicillin	Children older than 2 months with cough and fast or difficult breathing of less than 14 days receiving amoxicillin or cotrimoxazole for 5 days If the child is less than 2 month or had symptoms for 14 days or more, he/she should be referred If there are signs of severe pneumonia, he/she should be referred with IM chloramphenicol

^a^ARI timers are available in the Northern Region under the iCCM strategy to help diagnose suspected pneumonia. If severe pneumonia is suspected, the child must be referred to a CHPS compound or a Health Centre

^b^Nurses at CHPS compounds do not have ARI timers. The diagnosis is made based on clinical signs. If a severe pneumonia case is suspected, the children must be referred to a higher level of health facility. Some district hospitals, all regional hospitals and teaching hospitals have X-Rays to help diagnose pneumonia. Health centres, district hospitals, regional hospitals and teaching hospitals have laboratory facilities to help diagnose malaria, diarrhoea and pneumonia

### Data management and analysis

Data were double entered and validated using EpiData 3.1. Survey data processing and analysis was done using STATA 12. Initial data examination and prevalence estimates were obtained using tabulations adjusted for survey design. Pearson’s design based Chi square was used to test for associations. Survey logistic regression was used to obtain adjusted estimates.

**Fig. 1:**
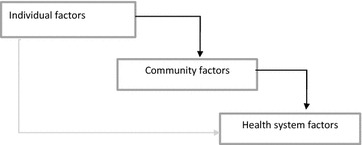
Simplified conceptual hierarchical framework for HBC and CHPS utilization

To explore the potential association between key outcome variables and potential predictors, the crude OR was obtained using univariate logistic regression, and the adjusted OR using multivariate analysis based on the framework below (Table 2; Fig. 1). The association of each factor (adjusted only for district) with the outcome was estimated. All individual factors whose association reached significance at p < 0.1 were included in a multivariate analysis. All factors that remained significantly associated with the outcome (p < 0.1) in this model were retained. The variables included in this model were the core group of individual variables. The same procedure was followed for community and health system factors. All remaining individual, community and health system variables were then combined in a multivariate analysis. All variables that remained significantly associated with the outcome (p < 0.05) in this model were retained in the final model. Two-way interactions were tested with all the variables retained in the final model.

**Table 2:** Variables of the framework for HBC and CHPS utilization

Category	Variable
Individual factors	Age of child
	Sex of child
	Age of care taker
	Education of care taker
	Household socio economic status
Community factors	Preventive messages sent by CBAs and CHPS
	Preventive messages sent by other sources
	Open hours (flexibility of time) of a CBA and CHPS to attend a child
Health system factors	Active NHIS card
	Distance to a health facility
	Type of closest facility
	Open hours (flexibility of time) of the closest facility

Principal components analysis was used to create socio-economic quintiles and compare outcomes across these quintiles. The variables used to generate the socioeconomic quintiles were ownership of the house, number of rooms, type of flooring, availability of electricity, radio, television, refrigerator, telephone, bicycle, motorcycle, car, canoe, tractor, source of water, type of sanitation, main source for cooking and number of people living in the household. The advantage of using a principal components analysis over the more traditional methods based on income and consumption expenditure is that it avoids many of the measurement problems like recall bias, seasonality and data collection time [33].

## Results

A total of 1356 interviews were conducted in the Volta and Northern Regions (685 and 671 respectively) (Table 3). Among the children included in the study, fever was the most prevalent reported symptom during the last 2 weeks [621/671 (90.9 %) in the Volta Region and 635/685 (94.4 %) in the Northern Region], followed by cough [408/671 (65.9 %) in the Volta Region and 334/685 (53.1 %) in the Northern Region] and diarrhoea [287/671 (49 %) in the Volta and 291/685 (42.7 %) in the Northern Region] (Table 4).

**Table 3:** Number of interviews conducted by district and region

Volta region		Northern region	
District name	Freq.	District name	Freq.
Hohoe municipality	115	Central Gonja	114
Jasikan	113	East Gonja	118
Ketu North	98	East Mamprusi	120
Krachi East	114	Saboba	110
Krachi West	121	Sawla-Tuna-Kalba	106
North Tongu	110	Tolon Kumbungu	117
Total interviews	671	Total interviews	685

**Table 4:** Prevalence of symptoms and care seeking behaviour by region

Indicator	Volta region	Northern region	N	%^b^
	N	%^b^		
Had fever during past 2 weeks^a^	621/671	90.9	635/685	94.4
Had diarrhoea during past 2 weeks	287/671	49.0	291/685	42.7
Had cough during past 2 weeks	408/671	65.9	334/685	53.1
Had suspected pneumonia during past 2 weeks	153/671	21.4	80/685	10.2
Sought care (for any of the three symptoms)	639/671	93.1	626/685	92.8
From CBA	90/671	17.3	8/685	1.0
From CHPS	61/671	11.8	228/685	31.3
From health centre	130/671	12.2	155/685	21.1
From hospital	153/671	24.2	83/685	13.0
From private clinic	19/671	4.0	25/685	7.3
From licensed chemical seller	153/671	19.5	88/685	14.9
From drug peddler	29/671	3.3	33/685	5.5
From traditional healer	0/671	0	6/685	0.6
From other providers	4/671	0.4	0/685	0
Care not sought	32/671	6.8	59/685	7.3
Not aware/don’t have CBA	213/671	29.8	314/685	40.6
Sought care in the first 24 h (for any of the three symptoms)	299/671	40.0	413/685	62.5
From CBA	58/90	56.0	6/8	79.9
From CHPS	22/61	36.3	163/228	76.9
From health centre	62/130	33.8	104/155	72.4
From hospital	60/153	45.7	54/83	59.0
From private clinic	4/19	20.6	12/25	47.8
From licensed chemical seller	74/153	40.8	58/88	59.1
From drug peddler	16/29	49.7	14/33	55.6
From traditional healer	0	0	2/6	43.2
From other providers	3/4	54.7	0	0
Sought care in the first 24 h in case of fever	278/621	40.2	385/635	62.5
Sought care in the first 24 h in case of diarrhoea	140/287	40.6	159/291	58.3
Sought care in the first 24 h in case of cough	178/408	39.4	188/334	54.9
Sought care in the first 24 h in case of suspected pneumonia	71/153	33.4	47/80	56.4
Sought care from appropriate provider (for any of the three symptoms)	609/671	89.6	587/685	86.4
Sought care from appropriate provider in first 24 h (for any of the three symptoms)	282/671	38.1	397/685	59.1

^a^Fever refers to hot body or chills

^b^Weighted estimates

### Utilization of HBC and CHPS strategies

Almost all respondents in both regions (93 %) indicated that they sought some form of care when the child’s symptoms started in the past 2 weeks preceding the survey, and more than 86 % did it from an appropriate provider (Table 4). Seeking care from an appropriate provider was not associated with the SES (p = 0.6 and p = 0.2 in the Volta and Northern Regions) but it was associated with having an active NHIS card in the Northern Region (p = 0.01).

About 30 % of carers visited a community-based health provider (HBC or CHPS) when their child had fever, cough or diarrhoea (29.1 and 32.3 % in the Volta and Northern Region). Although CHPS coverage was found to be similar in both regions (41 and 43 % of households have a CHPS as the closest health facility in the Volta and Northern Region) and the distance to the closest health facility is larger in the Northern Region (61 versus 45 % have a health facility at less than 1 h walking in the Volta and the Northern Region), HBC was more utilized than CHPS in the Volta Region (17.3 % of carers visited a CBA) and CHPS were much more used than HBC in the Northern Region (31.1 % of carers visited a CHPS) (Table 4).

Within regions the utilization of HBC and CHPS varied by districts (Table 5). HBC utilization in the Volta Region ranged from 35.3 % (95 % confidence interval (CI) 20.8–53) in Krachi East to 0.3 % (95 % CI 0.01, 0.9) in Jasikan (p = 0.001). In the Northern Region HBC utilization was generally very low and the percentage of carers reporting that they were not aware of CBAs or that they do not have CBAs in the community was higher than in the Volta Region [314/685 (40.6 %) versus 213/671 (29.8 %), respectively]. The utilization of CHPS in the Volta Region varied from 27.1 % (95 % CI 2.5, 84.3) in Krachi West to 2.5 % (95 % CI 0.3, 15.2) in Hohoe municipal (p = 0.2). In the Northern Region, the utilization of CHPS ranged from 56.5 (27.9, 81.2) in Saboba to 4.7 (2.4, 9.2) in Central Gonja (p = 0.004).

**Table 5:** Utilization of HBC and CHPS by district and region

Districts	Volta region^b^						Northern region						
	HBC			CHPS			HBC				CHPS		
	n/N	% (95 % CI)^a^	p	n/N	% (95 % CI)^a^	p	Districts	n/N	% (95 % CI)^a^	p	n/N	% (95 % CI)^a^	p
Jasikan	2/107	0.3 (0.01, 0.9)	0.001	13/107	11.3 (7.2, 15.5)	0.2	Sawla-Tuna-Kalba	1/97	1.1 (0.08, 13)	0.3	61/97	52.4 (22.3, 80.8)	0.004
Krachi East	22/111	35.3 (20.8, 53)		10/111	17.9 (11.5, 26.9)		Central Gonja	1/100	0.1 (0.009, 3.3)		8/100	4.7 (2.4, 9.2)	
Krachi West	23/119	12.7 (0.5, 78)		17/119	27.1 (2.5, 84.3)		Tolon Kumbungu	2/103	3.7 (1.6, 8.4)		24/103	20.0 (6.1, 48.7)	
Hohoe Mun.	10/111	10.9 (4.5, 23.9)		4/111	2.5 (0.3, 15.2)		East Gonja	0/112	0		46/112	22.6 (5.8, 57.7)	
Ketu North	12/91	12.2 (3.6, 33.7)		6/91	7.9 (5.1, 11.9)		Saboba	1/102	1.1 (0.3, 3.8)		55/102	56.5 (27.9, 81.2)	
North Tongu	21/100	19.9 (8.1, 41.2)		11/100	10.6 (1.4, 49.0)		East Mamprusi	3/112	2.4 (0.7, 7.5)		34/112	22.5 (14.6, 57.5)	
Total	90/639	18.5 (5.8, 45.7)		61/639	12.7 (6.7, 22.9)		Total	8/626	1.0 (0.2, 3.9)		228/626	33.7 (10.6,68.6)	

^a^Weighted estimates

^b^All these districts in the Volta Region implement only malaria management, although they have been trained for the management of the three diseases

Only 282/671 (38.1 %) of carers in the Volta Region and 397/685 (59.1 %) in the Northern Region reported that they sought care for their child from an appropriate provider the same day or the day after the onset of fever, diarrhoea or cough (Table 4). While children seeking care from a CBA within 24 h of onset of symptoms was significantly higher when compared with all other appropriate providers collated in the Volta Region [58/90, 56 % (95 % CI 48.7, 63.08) versus 224/519, 39.4 % (95 % CI 29.2, 50.5), p = 0.03], children seeking care from CHPS in the Northern Region also tended to do it more promptly when compared with other appropriate providers collated [163/227, 77.0 % (95 % CI 70.2, 82.7) versus 234/357, 63.6 % (95 % CI 50.2, 75.2), p = 0.02].

### Factors associated with HBC and CHPS utilization in the Volta Region

The final regression model showed that carers of sick children were more likely to visit a CBA if children were older than 6 months (adjusted OR 6–23 months 4.1, 95 % CI 3, 5.5; adjusted OR ≥24 months 4.1, 95 % CI 1.4, 11; p = 0.01), or if they lived further than 15 min walking distance to a health facility (adjusted OR health facility 15–30 min walking 36.9, 95 % CI 1.6, 805), p = 0.03; 30 min–1 h adjusted OR 61.8, 95 % CI 4.8, 788, p = 0.01; 1–2 h adjusted OR 85, 95 % CI 6.8, 1056, p = 0.01; ≥2 h adjusted OR 36.4 (1.5, 851), p = 0.03) (Additional file 1). Flexibility of time of the CBA to attend to a child had a borderline association with utilization of HBC: adjusted OR 14 (95 % CI 0.4, 417), p = 0.08. Carers from households in higher socio-economic quintiles were less likely to take their children to a CBA than those in the lowest socio-economic quintile (adjusted OR lower middle quintile 0.2, 95 % CI 0.08, 0.7, p = 0.03; adjusted OR upper middle quintile 0.3, 95 % CI 0.06, 1.4, p = 0.09; adjusted OR upper quintile 0.3, 95 % CI 0.01, 1.5, p = 0.08). No association with the middle SES quintile compared with the lower level was found.

No interaction was found between HBC utilization and any other variable. No factor was found to be associated with the utilization of CHPS compounds.

### Factors associated with HBC and CHPS utilization in the Northern Region

Due to low HBC utilization in the Northern Region (n = 8) it was not possible to look for predictors. With regards to CHPS utilization, carers having as the closest facility a health centre or a private clinic were less likely to go to a CHPS compound (adjusted OR health centre 0.01, 95 % CI 0.002, 0.08; adjusted OR private clinic 0.008, 95 % CI 0.001, 0.5, p = 0.02 (Additional file 2). No interaction was found.

### Appropriate treatment of malaria under the HBC and CHPS strategies

Regarding appropriate treatment of malaria, 19/77 (45.3 %) and 1/7 (14.9 %) of the children with fever that were taken to a CBA received ACT or were referred with artesunate to a health facility in the Volta and Northern Regions, respectively (Additional file 3); 18/77 (45.0 %) and 1/7 (14.9 %) in the Volta and Northern Regions received ACT and 12/77 (14.9 %) and 1/7 (14.9 %) in the Volta and Northern Regions received ACT within 24 h of the onset of symptoms. In Volta Region, some carers reported that they were prescribed amodiaquine monotherapy (6/78) and quinine (2/77) from CBAs. CBAs are not licensed to prescribe amodiaquine or quinine and amodiaquine should not be given as a monotherapy. However it is difficult to determine if carers were actually given amodiaquine in monotherapy or if carers reported “amodiaquine” as a short name of “artesunate-amodiaquine”. How these two drugs were supplied to CBAs was not clear: they may have been provided from the health facilities or CBAs may have purchased them at a local pharmacy for selling. However, carers did not report that they paid for these drugs.

**Fig. 2:**
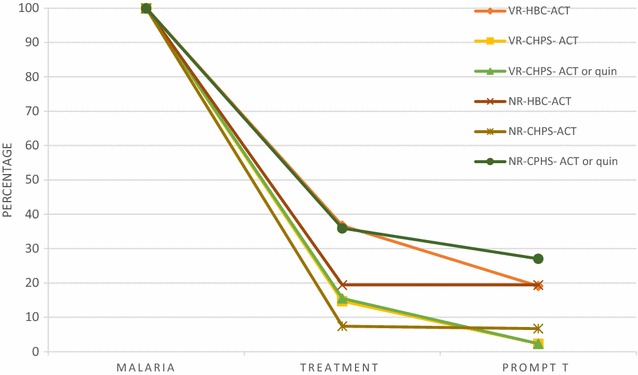
Case management of uncomplicated malaria under HBC and CHPS by region. (Uncomplicated malaria = cases tested positive or fevers when no test was conducted that were not referred). *VR* Volta region, *NR* Northern region, *quin* quinine, *T* treatment

In the case of the CHPS, 34/55 (65.3 %) and 86/209 (41.7 %) of the children with fever were tested for malaria in the Volta and Northern Regions. A high proportion of carers did not know the results of the test [9/37 (19.0 %) and 21/92 (24.9 %) in the Volta and Northern Regions respectively]. Of those who tested positive, 6/23 (20.8 %) and 14/67 (8.6 %) in the Volta and Northern Regions were given an ACT; 0/23 (0 %) and 13/62 (35.1 %) were given quinine (reserved for severe malaria cases that should be treated in hospital [34]) and 3/23 (22.3 %) and 2/62 (3.8 %) were given amodiaquine. When testing negative, only one case in the Volta Region was given ACT and none in the Northern Region. If considering together all uncomplicated malaria cases (those tested positive and fever cases without laboratory confirmation that were not referred), 7/40 (14.7 %) and 26/183 (7.4 %) in the Volta and Northern Regions received ACT (Fig. 2). If malaria cases treated with quinine are included, then the proportion of children appropriately treated increases especially in the Northern Region although still not satisfactory: 8/40 (15.5 %) and 57/183 (35.9 %) in the Volta and Northern Regions. Prompt treatment with ACT or quinine was also low: 1/40 (2.3 %) and 43/183 (27.3 %) in the Volta and Northern Regions respectively.

### Appropriate treatment of diarrhoea under the HBC and CHPS strategies

Of the children with diarrhoea that were taken to a CBA in the Volta Region, 4/38 (7.6 %) and 3/38 (5.7 %) received ORS or were referred and received ORS plus zinc or were referred, respectively.

In the case of the CHPS, only 6/31 (22.1 %) and 8/86 (5.6 %) of children with diarrhoea received ORS, 7/31 (31.3 %) and 4/86 (5.5 %) received zinc and 1/30 (0.3 %) and 0/86 (0 %) received ORS plus zinc in the Volta and Northern Regions, respectively.

### Appropriate treatment of suspected pneumonia under the HBC and CHPS strategies

Of the children with cough with fast or difficult breathing that were taken to a CBA, 7/25 (31.8 %) received amoxicillin or were referred in the Volta Region and 0/1 (0 %) received amoxicillin in the Northern Region. In the case of the CHPS, 1/9 (18.7 %) and 4/15 (33.0 %) in the Volta and in the Northern Region received amoxicillin or co-trimoxazole according to the protocol.

### Follow-up visits, referrals and second providers’ visits

National guidelines state the CBA must conduct a follow-up visit on the day after the first visit [16]. This follow-up visit was conducted for 38/88 (68.8 %) and 4/8 (32.3 %) of the cases in the Volta and Northern Regions. Artesunate suppositories were given along with a written referral in 2 of the 6 fever cases referred in the Volta Region and in none of the two cases in the Northern Region. No amoxicillin was given in case of referral because of suspected pneumonia in either region, and 2/8 (59.9 %) of the cough cases referred received amoxicillin in the Volta Region.

After visiting a CBA, 28/90 (42.4 %) and 4/8 (63.3 %) of the carers in the Volta and in the Northern Region went to a second provider. The main reason for this second visit in the Volta Region was children not getting better [24/28 (98.7 %)] while in the Northern Region the reported reasons were not getting better [2/4 (25.5 %)] and to get drugs [2/4 (74.5 %)] (Additional file 4). After visiting a CHPS, 14/61 (28.0 %) and 21/228 (7.9 %) in the Volta and in the Northern Region went to a second provider. The facilities more often visited were the licensed chemical sellers in the Volta Region to buy drugs [8/14 (50.4 %)] and health centres in the Northern Region because the child was not getting better [9/21 (23.8 %)].

### Users’ reported satisfaction

In general, users of HBC and CHPS in both regions reported that they were satisfied, although consistently more in the Volta Region (Table 6). Lack of affordability and availability of drugs were the factors more often reported as reasons for dissatisfaction with the services received.

**Table 6:** Users’ satisfaction after visiting CBA or a CHPS by region

	Very satisfied		Satisfied		Not sure		Not satisfied		Absolutely not satisfied	
	n/N	%	n/N	%	n/N	%	n/N	%	n/N	%
Volta Region										
CBA	29/89	32.6	52/89	58.4	0/89	0	6/89	6.7	2/89	2.2
CHPS	15/61	24.6	42/61	68.8	0/61	0	2/61	3.3	2/61	3.3
Northern Region										
CBA	2/8	16.1	4/8	30.1	1/8	13.6	0/8	0	1/8	39.9
CHPS	29/228	8.4	175/228	85.2	1/228	0.1	21/228	5.3	2/228	0.8

The main reason for not being satisfied when using HBC in the Volta Region was unavailability of drugs [5/8 (80.24 %)], while drugs not available, drugs not affordable and drugs not free [1/1, (100 %)] were the concerns in the Northern Region. It is important to note that three of the seven drugs (42 %) and 3/138 (2.1 %) given by the CBA in the Northern and the Volta Regions were sold to the carers.

Likewise, the main reason for not being satisfied when visiting a CHPS in the Northern Region was drugs not available (5/23, 39.1 %). CHPS users in the Volta Region reported a higher variety of reasons for not being satisfied (drugs not available, travel long distances, not time for seeking care and staff not giving information).

## Discussion

This study assessed the effectiveness of HBC and CHPS in terms of utilization, appropriate treatment given and satisfaction of carers of children under-five years of age with fever, diarrhoea or suspected pneumonia in the last 2 weeks prior to the interview.

### Utilization of HBC and CHPS

This study showed that seeking care from an appropriate provider in case of fever, diarrhoea or cough was high in both regions and higher than that found in previous surveys while being coherent with the increased trend on seeking care because of fever: the 2011 MICS survey showed that approximately 44 and 43 % of fever cases in under-fives in the Volta and Northern Regions sought care from an appropriate provider at any time [28]; the 2013 LQAS survey showed that 60 % of fever cases in under-fives in the Northern Region sought care at any time and 30 % in the first 24 h of onset of symptoms [36] and in the 2014 DHS 73.6 and 83.6 % of fever cases in the Volta and Northern Region sought care at any time [37]. It should be noted that data from the LQAS survey is not representative of the Northern Region as they purposively selected 10 districts out of 20. The MICS and the DHS surveys, use a similar sampling methodology but their sample size of children under-five presenting with fever was between three and 11 times smaller than this study.

The total utilization of community-based interventions was similar and slightly higher in the Northern Region when compared with the Volta Region (32.3 versus 29.1 %). However utilization of HBC versus CHPS was different: HBC was more used in the Volta Region while CHPS was more used in the Northern Region.

The HBC utilization found in this study was similar to the 2013 LQAS survey in the Northern Region (95 % CI 0.7, 6.5 %). However, another study conducted in one district of the Ashanti and Volta Regions in 2008 [26] showed higher HBC utilization (more than 68 and 75 % respectively used HBC a year after the HBC implementation). When compared with other evaluations conducted in Uganda [38], which was a quasi-experimental study before and after 18 months implementation of HBC, and in Burkina Faso, which was a cross sectional study conducted before and after 1 year implementation of HBC, a higher utilization of HBC (25 and 56 % respectively) was found. Two reasons could explain this higher HBC utilization. Firstly, due to differences between districts: the study in the Ashanti and Volta Region only focussed in one district per region and this current study has shown the variation of HBC utilization among districts specially in the Volta Region). Secondly, due to differences between research projects and real world implementation: the length of the projects is generally shorter and the quality and intensity of supervision is usually better in research projects than in routine programme implementation. For example, the HBC strategy in the Northern Region was being implemented for about 8 years before the survey and for 2 years in the Volta Region. Longer time implementing the strategy might bring expertise but also tiredness of the CBAs and the supervisor, stock-out of drugs and the need for CBA replenishment and training. The lower effectiveness of implementation of an intervention in the “real world” as compared to that found in research projects is already being discussed and addressed through the Implementation Research [39–41], which aims to bridge the implementation gap between knowledge and action.

Large differences in HBC utilization were observed between the Volta and Northern Regions. However, the HBC strategy in the two regions started at different times and includes different interventions. In the Northern Region, HBC started in 2007 first addressing malaria cases, and in 2010 the management of diarrhoea and suspected pneumonia cases were included with the technical and financial support of UNICEF. The HBC in the Volta Region started in 2012 and includes only drugs for the management of malaria cases with the financial support of the GFATM, while diarrhoea and suspected pneumonia cases should be referred for further treatment. Therefore, one could argue that a higher HBC utilization in the Northern Region would be expected as a wider range of conditions are treated by the CBAs compared with Volta Region (which was not the case). Considering that this study was conducted in communities where according to policy HBC is being implemented, it is surprising that 30 % of carers in the Volta Region and 41 % in the Northern region indicated that they were not aware of the presence of CBAs or they did not have CBAs in the community. During informal communications with CBA and community chiefs while conducting the survey, the field team was informed that some CBAs travelled and no one had replaced them yet, others stopped working as they did not have drugs to work with and some CBAs were known in one area of the community while not in another area, suggesting that social and personal issues might also affect the knowledge and the utilization of the CBA services. Therefore, sociocultural issues, stock out of CBA drugs or high turnover of CBAs could explain the lower utilization of HBC in the Northern Region as it has also been reported in other studies in Ghana and elsewhere as a barrier to implementation [27, 38]. A further qualitative study might help to understand causes of the low HBC utilization in the Northern Region.

With respect to the Volta Region, HBC utilization was not associated with living far from a CHPS or with low flexibility of CHPS for attending patients. The HBC strategy in the Volta Region was found to be coherent with the guidelines in terms of not treating children under 6 months, is reaching the poorest in coherence with its intention of being a “pro-poor” intervention and it is more used when there is no health facility close to the house. It is worth noting that a study in Uganda in 2007 [38] concluded that HBC was less likely to reach the poorest and the authors could not explain why. Two other studies in Uganda and Zambia found that proximity to a health facility is a deterrent against HBC utilization [42, 43] and another one found that HBC is not cost-effective in the context of proximity to a health facility [11]. For future planning and considering only the therapeutic component of the HBC (which is the one evaluated in this paper), implementation of HBC should consider to target areas without a health facility (as it was with the strategy of the NMCP in the Volta Region).

With regards to CHPS utilization, proximity to a CHPS was found to be associated to CHPS utilization in the Northern Region (and not in the Volta Region, were carers chose a provider based on different criteria). The percentage of carers visiting a CHPS compound was higher than the results of the LQAS survey (between 6 and 10 % of carers visited a CHPS compound when their child was sick). No other comparable studies on CHPS utilization were found in the literature to contrast these results.

Carers visiting a CBA in the Volta Region and visiting a CHPS in the Northern Region did it more promptly when compared with other providers. When diagnosed with malaria, children visiting a CBA also received ACT more promptly than when visiting any other provider in the Volta Region (22.4 versus 3.8 %, p = 0.05). Prompt treatment received from a CBA was reported in other studies conducted in Rwanda [44], Uganda [38, 45], Ghana [26, 27], Nigeria [26], Burkina Faso [27, 46], Tanzania [47], Ethiopia [27] and Malawi [27]. Most of these studies looked at the performance of HBC at a point in time or in before-after cross-sectional studies. Only the two studies in Uganda (an RCT and a quasi-experimental study) were designed to test for a difference in prompt treatment seeking between HBC and standard treatment and their results were similar: 62 versus 37 %, p = 0.0001 [45] and a significant difference at post intervention (12.3 %, p = 0.05) [38].

### Appropriate treatment

Both HBC and CHPS failed in reaching the target of treating 100 % of eligible children with ACT, amoxicillin and/or ORS + zinc. This worrying fact should question the local health authorities particularly on the adequacy of the drug supply chain. Acknowledging the difficulty of interpreting the HBC figures due to the low numbers of carers visiting a CBA in the Northern Region, it seems that HBC was more used and performed better in the Volta Region when compared with the Northern Region while CHPS in the Northern Region were more used and performed better than in the Volta Region.

More malaria cases were treated with quinine (reserved for complicated cases) than with ACT in the CHPS of Northern Region. As only between 0 and 11 % of cases seen in health facilities in Ghana are complicated cases [48, 49] a possible explanation for the frequent use of quinine could be stock-outs of drugs The percentage of uncomplicated malaria cases treated with ACT in this study is lower than that found in other studies [23, 26, 27]. The source of information (CBA records versus carers’ information) could contribute to the different results. It is important to note that both, CBA records and carers’ information are not the gold standard for collecting this type of information, which is considered to be the direct observation of CBA work [14]. CBA registers may suffer from inaccurate or incomplete reporting and household surveys may suffer from recall bias and misunderstanding. However, the few studies that collected data from both sources found similar results [14, 26, 27]. As mentioned before, other factors that could explain differences in performance are better supervision, better supply of drugs with the involvement of the research teams, as well as a shorter duration of the research projects when compared to program implementation. Another study conducted in Burkina Faso [46] with less external supervision or anti-malarial supply had similar results to this study (54 % of the febrile children received ACT from a CBA).

With regards to diarrhoea management, a common finding was the low percentage of cases correctly treated and children receiving either ORS or zinc, but not both at the same time. The LQAS survey in the Northern Region also found a low proportion of diarrhoea cases treated with ORS and zinc when visiting a CBA or a CHPS, suggesting that it might be due to stock-outs of drugs. CBAs are provided with drugs during the monthly community welfare clinics conducted by CHPS’ or health centres’ nurses. However, this integration of services does not seem to cover all drug needs. Results of the 2011 MICS and the 2014 DHS also showed a low proportion of diarrhoea cases correctly treated (2011 MICS: 32 and 30.1 % of diarrhoea cases received ORS and 0 and 0.2 % received zinc from an appropriate provider excluding pharmacies in the Volta and Northern Regions respectively; 2014 DHS: 41.3 and 48.7 % of diarrhoea cases received ORS and 0 and 5 % received zinc from an appropriate provider excluding pharmacies in the Volta and Northern Regions). The low coverage of ORS and almost negligible use of zinc to treat diarrhoea cases was also reported by Gill et al. [50]. In their paper about bottlenecks, barriers and solutions to the low implementation of effective measures to reduce childhood pneumonia and diarrhoea deaths in low and middle income countries, they stated that the main bottlenecks for diarrhoea appropriate treatment were concentrated in downstream areas related to provision of ORS and zinc in the community.

The study results regarding the appropriate treatment of suspected pneumonia cases cannot be compared with the 2011 MICS and the 2014 DHS as these surveys did not report on this indicator based on the different providers visited. Another three studies conducted in Africa on HBC [43, 51, 52] showed a better performance (between 63 and 98 % of suspected cases received amoxicillin). However, two of them [43, 51] used a different methodology to diagnose suspected pneumonia cases (registries and direct observation of CBA as opposed to carers’ reports) and another one used carers reported symptoms but in the context of a cluster randomized trial.

Finally, there is the need to reflect on the fact that some cases received amoxicillin, ORS or zinc when visiting a CBA in the Volta Region (as the GFATM is only supporting ACT). It seems feasible to believe that CBAs still had some of these drugs distributed in 2013 in stock, or CBAs bought these drugs to be distributed among sick children.

### CBA’s referrals and second visit to health providers

This study showed a higher proportion of carers that sought care elsewhere after visiting a CBA than the study in Dangme West district [23] (where only 3.9 % of the carers sought care elsewhere). Since for the HBC strategy in the Volta Region CBAs were expected to refer diarrhoea and suspected pneumonia cases, one would expect a higher proportion of carers seeking care elsewhere in the Volta Region. However, 63 and 42 % of carers in the Northern and Volta Region sought care elsewhere after visiting a CBA because of unavailability of drugs and children not getting better. This is coherent with the LQAS survey where only 16 % of the CBAs had ACT, ORS, Zinc and amoxicillin on the day of the survey in the Northern Region. With regards to the HBC in the Volta Region, it is important that iCCM coordinators emphasize on the importance of referral with a form, as seeking care elsewhere can be seen as a failure of the program while referral can be interpreted as appropriate management of cases.

The low coverage of appropriate treatment found should make us reflect upon the new “Integrated community case management guidelines” which will include pregnancy and neonatal care, nutrition in under-fives and the inclusion of RDT for the diagnosis of malaria. Before adding more components to the HBC strategy, adherence to protocol through ensuring availability of drugs, adequate supervision and continuous replacement of CBAs must be ensured.

### Users’ satisfaction

Lack of availability and affordability of drugs were the main factors for carer dissatisfaction of services in both regions. Therefore, emphasis must be given to avoiding drug stock outs. Also, a reflexion must be undertaken about carers paying CBAs for drugs. Although paying for CBAs’ drugs can be a strategy to retain CBAs in their task, carers valued free drugs as a positive element of the HBC strategy. Secondly, it is a contradiction with the NHIS which established free treatment for children under-five. Thirdly, because this practice is not considered in the guidelines, which only states “to ensure that cost of iCCM would not be a barrier to accessing treatment, drugs should be given to clients at no cost or National Health Insurance Scheme (NHIS) may cover all drugs”.

### Limitations of the study

Response rate was very high for the survey with no refusals to participate. The variables described in the study represent only the population of sick children during the last 2 weeks prior the interview and not the whole population.

The study looked at programme implementation against guidelines of the national programme. Comparison between north and south was descriptive, understanding that regions are different from the cultural and epidemiological point of view and without directly comparing malaria HBC with integrated HBC. Different epidemiological burden would not be expected to influence results, as the target population was children with symptoms. However, finding these children when doing the data collection was easier in the Northern Region as the prevalence of the three diseases was higher. As this is a cross-sectional study, no reference to causality can be made, only association among variables.

Results are based on responses of carers of children under-five. Morbidity data collected is subjective as it is based on a mother’s perception of illness and their understanding about their children’s disease and the treatment given, with no validation of their responses by for example comparison with that of the CBAs by looking at the CBAs forms or CHPS registries. Therefore, interpreting results particularly related to suspected pneumonia must be done with caution as fast breathing, chest in drawing or noisy breathing can be perceived differently by the carers and the provider and therefore, treated differently. The same is the case with diagnostic procedures and treatment given and understood. A patient could have been given “artesunate-amodiaquine” but referred to have received “amodiaquine”. Or the patient might not remember the name of the drug, even with the help of the drugs pictures that were taken to the field to conduct the survey. However, the use of carers’ reports to classify malaria, diarrhoea and suspected pneumonia has been used in the MICS, DHS and other studies [52]. In addition, two studies on anti-malarial use and dosage using both sources of data (HBC records and carers’s reports) showed similar results [26, 27]. Finally if some children were misclassified (for example being attributed with one symptom while they do not have it), this is not likely to have introduced a differential bias between HBC and CHPS.

Some of the results had large confidence intervals even though the formula used to calculate the sample size was adequate. The clustering of indicators by district was larger than expected and therefore a bigger design effect could have been more appropriate (Design effect = 2 instead of 1.5). As a result, the sample size was small for some indicators.

## Conclusions

HBC was more used in the Volta Region while CHPS was more used in the Northern Region. HBC utilization was almost non-existent in the Northern Region. Poorer children, children older than 6 months and those living far from a health facility were more likely to use HBC in the Volta Region. HBC contributed to prompt treatment of fevers in the Volta Region.

Appropriate treatment for the three diseases was low in the HBC and CHPS areas, in the Volta and Northern Regions. Carers were satisfied with the services received. Lack of availability and affordability of drugs were the factors more reported as cause of dissatisfaction.

More efforts should be made in the provision of drugs, ensuring that CBAs are in service and in monitoring the CBA and CHPS performance, especially if more components are to be included in the HBC strategy. A well-functioning integration of services might help to improve provision of drugs and supervision. Sustainability of HBC needs to be addressed. A cost-effectiveness study of the HBC compared with CHPS might help to guide decisions on future financing and motivation to CBA in Ghana.

## Additional files

10.1186/s12936-016-1380-9 Unadjusted and adjusted predictors of HBC and CHPS utilization in the Volta Region.
10.1186/s12936-016-1380-9 Unadjusted and adjusted predictors of CHPS utilization in the Northern Region.
10.1186/s12936-016-1380-9 Proportion of symptomatic children receiving appropriate treatment under HBC and CHPS by region.
10.1186/s12936-016-1380-9 Places and reasons for seeking care elsewhere after visiting CBA or a CHPS by region.

## Abbreviations

ACT: artemisinin-based combination therapy
ARI: acute respiratory infection
CBAs: community-based agents
CHPS: community-based health planning services
DHS: demographic health survey
GFATM: global fund for AIDS, tuberculosis and malaria
GHS: Ghana health service
iCCM: integrated community case management
IMCI: integrated management of childhood illness
LQAS: lot quality assurance sampling
MICS: multiple indicator cluster survey
NHIS: National health insurance scheme
NMCP: National malaria control programme
ORS: oral rehydration salts
RDT: rapid diagnostic test
UNICEF: United Nations Children’s Fund
