# Supplementary material for: Integrated community case management and community-based health planning and services: a cross sectional study on the effectiveness of the national implementation for the treatment of malaria, diarrhoea and pneumonia
Source: Malar J. 2016 Jul 2;15:340. doi: 10.1186/s12936-016-1380-9 (PMC4930600; doi:10.1186/s12936-016-1380-9)
Supplement: Supplementary file 1 — 10.1186/s12936-016-1380-9 Unadjusted and adjusted predictors of HBC and CHPS utilization in the Volta Region. [file 12936_2016_1380_MOESM1_ESM.docx]

**Additional file 1. Unadjusted and adjusted predictors of HBC and CHPS utilization in the Volta Region**

|  | **HBC** | | | | | | **CHPS** | | | | | |
| --- | --- | --- | --- | --- | --- | --- | --- | --- | --- | --- | --- | --- |
| **Potential predictors** | **Unadjusted** | | | | **Adjusted** | | **Unadjusted** | | | **Adjusted** | | |
|  | **n/N** | **%*** | **OR (95% CI)** | **P **** | **OR (95% CI)** | **P** | **n/N** | **%*** | **OR (95% CI)** | **P **** | **OR (95% CI)** | **P** |
| **Sex child** |  |  |  |  |  |  |  |  |  |  |  |  |
| Female | 38/273 | 12.5 | 1.0 | 0.3 |  |  | 20/273 | 8.2 | 1.0 | 0.1 |  |  |
| Male | 43/335 | 18.1 | 1.5 (0.4, 5.1) |  |  |  | 37/335 | 15.3 | 2.0 (0.6, 6.4) |  |  |  |
| **Age group** |  |  |  |  |  |  |  |  |  |  |  |  |
| <6 months | 4/48 | 13.1 | 1.0 | 0.07 | 1.0 | 0.01 | 6/48 | 24.6 | 1.0 | 0.2 |  |  |
| 6-23 months | 37/244 | 24 | 2.3 (1.4, 3.7) |  | 4.1 (3, 5.5) |  | 25/244 | 13.2 | 0.4 (0.1, 1.4) |  |  |  |
| >=24 months | 49/347 | 16.4 | 1.7 (0.3, 7.8) |  | 4.1 (1.4, 11) |  | 30/347 | 10.3 | 0.3 (0.1, 1.0) |  |  |  |
| **Marital status** |  |  |  |  |  |  |  |  |  |  |  |  |
| Single | 5/44 | 5.1 | 1.0 | 0.6 |  |  | 5/44 | 13.4 | 1.0 | 0.3 |  |  |
| Married/co-habited | 82/548 | 19.6 | 5.8 (0.6, 56) |  |  |  | 54/548 | 14.1 | 0.9 (0.1, 8.1) |  |  |  |
| Separated/divorced | 2/34 | 1.8 | 0.5 (0.0, 6.5) |  |  |  | 1/34 | 0.1 | 0.008 (0.0001, 0.3) |  |  |  |
| Widowed | 1/13 | 53.8 | 24 (0.3, 1567) |  |  |  | 1/13 | 0.4 | 0.02 (0.0004, 1.08) |  |  |  |
| **Age respondent** |  |  |  |  |  |  |  |  |  |  |  |  |
| <20 years | 3/33 | 8.4 | 1.0 | 0.9 |  |  | 1/33 | 1.7 | 1.0 | 0.6 |  |  |
| 20-29 years | 36/281 | 20.7 | 1.2 (0.04, 33) |  |  |  | 27/281 | 13.5 | 6.9 (0.2, 198) |  |  |  |
| 30-39 | 34/215 | 13.9 | 0.7 (0.01, 33) |  |  |  | 25/215 | 14.3 | 7.2 (0.2, 176) |  |  |  |
| >=40 years | 15/106 | 23.6 | 1.5 (0.03, 66) |  |  |  | 7/106 | 8.5 | 4.3 (0.06, 292) |  |  |  |
| **Education of care taker** |  |  |  |  |  |  |  |  |  |  |  |  |
| None formal | 51/243 | 30.3 | 1.0 | 0.5 |  |  | 27/213 | 16.5 | 1.0 | 0.1 |  |  |
| Primary | 14/142 | 6.6 | 0.2 (0.09, 0.9) |  |  |  | 22/142 | 15.3 | 1.3 (0.1, 9.4) |  |  |  |
| Middle/secondary | 24/246 | 10.2 | 0.4 (0.08, 2.1) |  |  |  | 12/246 | 5.8 | 0.4 (0.06, 2.5) |  |  |  |
| Technical/Tertiary | 1/8 | 4.9 | 0.3 (0.003, 41) |  |  |  | 0/8 | 0 | - |  |  |  |
| **Socioeconomic status** |  |  |  |  |  |  |  |  |  |  |  |  |
| Lower | 20/129 | 41.7 | 1.0 |  | 1.0 |  | 24/129 | 16.5 | 1.0 |  |  |  |
| Lower middle | 21/122 | 14.7 | 0.2 (0.07, 0.7) | 0.03 | 0.2 (0.08, 0.7) | 0.03 | 14/122 | 18.8 | 1.3 (0.8, 2.1) | 0.14 |  |  |
| Middle | 23/126 | 13 | 0.2 (0.03, 23) | 0.15 | 0.3 (0.04, 3.3) | 0.2 | 10/126 | 10.0 | 0.8 (0.1, 4.4) | 0.7 |  |  |
| Upper Middle | 16/125 | 10.6 | 0.2 (0.004, 1.4) | 0.08 | 0.3 (0.06, 1.4) | 0.09 | 5/125 | 5.9 | 0.4 (0.01, 17.4) | 0.5 |  |  |
| Upper | 9/128 | 6.3 | 0.1 (0.01, 1.5) | 0.08 | 0.1 (0.01, 1.5) | 0.08 | 7/128 | 9.1 | 0.7 (0.09, 6.7) | 0.7 |  |  |
| **CBA accessibility** |  |  |  |  |  |  |  |  |  |  |  |  |
| Not flexible | 4/82 | 4.3 | 1.0 | 0.09 | 1.0 | 0.08 | 3/82 | 1.9 | 1.0 | 0.2 |  |  |
| Flexible | 86/299 | 36.6 | 14 (0.3, 537) |  | 14 (0.4, 417) |  | 29/299 | 17.6 | 10.8 (0.6, 182) |  |  |  |
| Not aware/don’t have CBA | 0/202 | 0 | - |  |  |  | 29/202 | 12.9 | 7.4 (0.3, 159) |  |  |  |
| Don’t know | 0/56 | 0 | - |  |  |  |  |  |  |  |  |  |
| **Receiving preventive messages from CBA/CHPS compounds** |  |  |  |  |  |  |  |  |  |  |  |  |
| No | 30/479 | 6.9 | 1.0 | 0.003 | 1.0 | 0.9 | 13/427 | 3.9 | 1.0 | 0.4 |  |  |
| Yes | 40/87 | 58.7 | 12.2 (4.9, 30.2) |  | 0.9 (0.9. 1) |  | 42/139 | 29.3 | 1.2 (0.5, 3.0) |  |  |  |
| **Active NHIS** |  |  |  |  |  |  |  |  |  |  |  |  |
| Yes | 65/452 | 16.4 | 1.0 | 0.3 |  |  | 42/452 | 11.9 | 1.0 | 0.5 |  |  |
| No | 25/187 | 23.8 | 1.4 (0.4, 4.4) |  |  |  | 19/187 | 14.6 | 1.2 (0.3, 4.1) |  |  |  |
| **Distance to facility (including CHPS)** |  |  |  |  |  |  |  |  |  |  |  |  |
| Less than 15 min walking | 4/50 | 0.4 | 1.0 |  | 1.0 |  | 14/143 | 11.2 | 1.0 |  |  |  |
| Between 15 min- 30 walking | 6/92 | 8.9 | 36 (0.7, 1793) | 0.04 | 36.9 (1.6, 805) | 0.03 | 16/180 | 9.5 | 0.7 (0.2, 2.6) | 0.5 |  |  |
| Between 30 min-1 hour walking | 38/180 | 20.8 | 58.8 (5.3, 645) | 0.01 | 61.8 (4.8, 788) | 0.01 | 23/191 | 16.2 | 1.1 (0.3, 3.4) | 0.7 |  |  |
| Between 1 and 2 hours walking | 31/191 | 26.6 | 69.1 (5, 950) | 0.01 | 85 (6.8, 1056) | 0.01 | 6/69 | 16.5 | 1.2 (0.1, 12.2) | 0.7 |  |  |
| More than 2 hours walking | 11/118 | 16.4 | 29.5 (0.8, 1044) | 0.05 | 36.4 (1/5, 851) | 0.03 | 2/49 | 10.7 | 0.8 (0.03, 23) | 0.9 |  |  |
| **Health facility accessibility/ CHIPS accessibility** |  |  |  |  |  |  |  |  |  |  |  |  |
| Not flexible | 6/81 | 19.9 | 1.0 | 0.5 |  |  | 15/46 | 29.5 | 1.0 | 0.9 |  |  |
| Flexible | 81/535 | 17.9 | 1.1 (0.6, 2.1) |  |  |  | 40/110 | 27.4 | 1.0 (0.4, 2.1) |  |  |  |
| **Closest facility** |  |  |  |  |  |  |  |  |  |  |  |  |
| CHPS | 22/165 | 27.2 | 1.0 | 0.6 |  |  | 55/165 | 26.0 | 1.0 | 0.1 |  |  |
| Health Centre | 27/275 | 14.8 | 0.4 (0.09, 1.7) |  |  |  | 2/275 | 0.7 | 0.02 (0.0009, 0.5) |  |  |  |
| District Hospital | 36/167 | 10.7 | 0.7 (0.06, 7.4) |  |  |  | 4/167 | 5.9 | 0.2 (0.01, 2.6) |  |  |  |
| Regional hospital | 0/3 | 0 | - |  |  |  | 0/3 | 0 | - |  |  |  |
| Private clinic | 5/24 | 11.1 | 0.5 (0.02,11.4) |  |  |  | 0/24 | 0 | - |  |  |  |
| Other | 0/5 | 0 | - |  |  |  | 0/5 | 0 | - |  |  |  |
| *Weighted estimates. ** Overall P-value not available for all variables due to sparse data within stratified categories' | | | | | | | | | | | | |
