# Supplementary material for: Integrated community case management and community-based health planning and services: a cross sectional study on the effectiveness of the national implementation for the treatment of malaria, diarrhoea and pneumonia
Source: Malar J. 2016 Jul 2;15:340. doi: 10.1186/s12936-016-1380-9 (PMC4930600; doi:10.1186/s12936-016-1380-9)
Supplement: Supplementary file 2 — 10.1186/s12936-016-1380-9 Unadjusted and adjusted predictors of CHPS utilization in the Northern Region. [file 12936_2016_1380_MOESM2_ESM.docx]

| **Additional file 2. Unadjusted and adjusted predictors of CHPS utilization in the Northern Region** | | | | | | |
| --- | --- | --- | --- | --- | --- | --- |
|  | **CHPS** | | | | | |
| **Potential predictors** | **Unadjusted** | | | | **Adjusted** | |
|  | **n/N** | **%*** | **OR (95% CI)** | **P **** | **OR (95% CI)** | **P** |
| **Sex child** |  |  |  |  |  |  |
| Female | 103/267 | 35.0 | 1.0 | 0.1 |  |  |
| Male | 101/278 | 36.1 | 1.2 (0.8, 1.8) |  |  |  |
| **Age group** |  |  |  |  |  |  |
| <6 months | 20/51 | 34.6 | 1.0 | 0.5 |  |  |
| 6-23 months | 84/228 | 29.6 | 0.5 (0.1, 1.9) |  |  |  |
| >=24 months | 124/347 | 36.5 | 0.7 (0.09, 5.2) |  |  |  |
| **Marital status** |  |  |  |  |  |  |
| Single | 4/9 | 27.4 | 1.0 | 0.8 |  |  |
| Married/co-habited | 219/597 | 33.8 | 0.6 (0.08, 5.5) |  |  |  |
| Separated/divorced | 2/4 | 57.6 | 0.8 (0.03, 18.6) |  |  |  |
| Widowed | 3/15 | 36.1 | 0.4 (0.04, 4.3) |  |  |  |
| **Age respondent** |  |  |  |  |  |  |
| <20 years | 8/24 | 25.6 | 1.0 | 0.6 |  |  |
| 20-29 years | 108/259 | 36.4 | 2.1 (0.6, 6.6) |  |  |  |
| 30-39 | 76/230 | 30.7 | 1.5 (0.5, 4.9) |  |  |  |
| >=40 years | 36/111 | 35.5 | 1.8 (0.7, 4.7) |  |  |  |
| **Education of care taker** |  |  |  |  |  |  |
| None formal | 183/510 | 35.4 | 1.0 | 0.4 |  |  |
| Primary | 21/56 | 13.8 | 0.4 (0.1, 1.0) |  |  |  |
| Middle/secondary | 22/51 | 44 | 1 (0.3, 3.2) |  |  |  |
| Technical/Tertiary | 2/8 | 2 | 0.01 (0.0002, 1.1) |  |  |  |
| **Socioeconomic status** |  |  |  |  |  |  |
| Lower | 38/119 | 39.8 | 1.0 |  |  |  |
| Lower middle | 43/125 | 36.6 | 1.0 (0.4, 2.1) | 1 |  |  |
| Middle | 49/120 | 37.4 | 1.0 (0.1, 7.4) | 0.9 |  |  |
| Upper Middle | 50/122 | 29.8 | 0.6 (0.1, 3.8) | 0.4 |  |  |
| Upper | 42/128 | 12.1 | 0.1 (0.003, 10.1) | 0.2 |  |  |
| **CBA accessibility** |  |  |  |  |  |  |
| Flexible | 93/212 | 48.3 | 1.0 | 0.2 |  |  |
| Not flexible | 38/100 | 35.1 | 0.5 (0.001, 17.9) |  |  |  |
| Not aware/don’t have CBA | 87/292 | 19.5 | 0.2 (0.06, 1.1) |  |  |  |
| **Receiving preventive messages from CHPS compounds** |  |  |  |  |  |  |
| No | 61/354 | 16.1 | 1.0 | 0.2 |  |  |
| Yes | 150/194 | 77.2 | 2.2 (0.3, 14.6) |  |  |  |
| **Active NHIS** |  |  |  | 0.9 |  |  |
| No | 55/204 | 25.3 | 1.0 |  |  |  |
| Yes | 173/422 | 38.9 | 1 (0.5, 1.9) |  |  |  |
| **Distance to facility** |  |  |  |  |  |  |
| Less than 15 min walking | 166/283 | 54.2 | 1.0 |  |  |  |
| Between 15 min- 30 walking | 23/113 | 25.1 | 0.3 (0.09, 1.2) | 0.07 | 0.2 (0.01, 3.0) | 0.1 |
| Between 30 min-1 hour walking | 23/103 | 27.1 | 0.4 (0.1, 1.4) | 0.1 | 1.1 (0.04, 26.2) | 0.9 |
| Between 1 and 2 hours walking | 7/50 | 12.8 | 0.1 (0.01, 1.20 | 0.06 | 0.3 (0.001, 71) | 0.5 |
| More than 2 hours walking | 9/71 | 8.1 | 0.1 (0.03, 0.7) | 0.03 | 0.6 (0.02, 16.0) | 0.7 |
| **CHPS accessibility** |  |  |  |  |  |  |
| Not flexible | 18/34 | 46.5 | 1.0 | 0.3 |  |  |
| Flexible | 205/293 | 76 | 2.6 (0.1, 51.2) |  |  |  |
| **Closest facility** |  |  |  |  |  |  |
| CHPS | 223/327 | 73.9 | 1.0 | 0.02 | 1.0 | 0.02 |
| Health Centre | 4/216 | 4.3 | 0.01 (0.003, 0.09) |  | 0.01 (0.002, 0.08) |  |
| District Hospital | 0/42 | 0 | - |  |  |  |
| Regional hospital | 0/0 | 0 | - |  |  |  |
| Private clinic | 1/26 | 4.4 | 0.008 (0.0001, 0.6) |  | 0.008 (0.0001, 0.5) |  |

*Weighted estimates. ** Overall P-value not available for all variables
