# Supplementary material for: Integrated community case management and community-based health planning and services: a cross sectional study on the effectiveness of the national implementation for the treatment of malaria, diarrhoea and pneumonia
Source: Malar J. 2016 Jul 2;15:340. doi: 10.1186/s12936-016-1380-9 (PMC4930600; doi:10.1186/s12936-016-1380-9)
Supplement: Supplementary file 3 — 10.1186/s12936-016-1380-9 Proportion of symptomatic children receiving appropriate treatment under HBC and CHPS by region. [file 12936_2016_1380_MOESM3_ESM.docx]

| **Additional file 3. Proportion of symptomatic children receiving appropriate treatment under the HBC and CHPS by region.** | | | | |
| --- | --- | --- | --- | --- |
| **Indicator** | **Volta Region** | | **Northern Region** | |
|  | **N** | **%*** | **N** | **%*** |
| **HBC** | | | | |
| Fever received ACT or referred with artesunate | 19/77 | 45.3 | 1/7 | 14.9 |
| Fever received ACT | 18/77 | 45.0 | 1/7 | 14.9 |
| Fever received prompt ACT | 12/77 | 14.9 | 1/7 | 14.9 |
| Fever prescribed 3 days of ACT | 16/77** | 44.9 | 0/7*** | 0 |
| Fever received 3 days ACT | 14/77 | 40.7 | 0/7 | 0 |
| Fever received Amodiaquine | 6/78 | 9.2 | 0/7 | 0 |
| Fever received quinine | 2/77 | 0.3 | 0/7 | 0 |
| Diarrhoea received ORS | 2/38 | 2.2 | 1/4 | 35.6 |
| Diarrhoea received ORS or was referred**** | 4/38 | 7.6 | NA |  |
| Diarrhoea received zinc | 3/38 | 16.3 | 1/4 | 8.9 |
| Diarrhoea received zinc or was referred**** | 6/38 | 22.1 | NA |  |
| Diarrhoea received zinc for 15 days | 0/38 | 0 | 0/4 | 0 |
| Diarrhoea received zinc and ORS or was referred**** | 3/38 | 5.7 | NA |  |
| Suspected pneumonia received amoxicillin | 6/25 | 14.1 | 0/1 | 0 |
| Suspected pneumonia received amoxicillin or was referred**** | 7/25 | 31.8 | NA |  |
| Follow up visit | 38/88 | 68.8 | 4/8 | 32.3 |
| Referred with a referral form | 5/9 | 62.9 | 0/2 | 0 |
| Referred with artesunate suppository in case of fever | 2/6 | 6.9 | 0/2 | 0 |
| Referred with amoxicillin in case of cough | 2/8 | 59.9 | 0/2 | 0 |
| Referred with amoxicillin in case of suspected pneumonia | 0/3 | 0 | 0/1 | 0 |
| Referred with amoxicillin in case of severe pneumonia signs | 0/2 | 0 | 0/2 | 0 |
| **CHPS** | | | | |
| Fever cases tested for malaria | 34/55 | 65.3 | 86/209 | 41.7 |
| Test positive for malaria (some cases with no fever were tested) | 23/37 | 62.1 | 67/92 | 71.2 |
| - Received ACT | 6/23 | 20.8 | 14/67 | 8.6 |
| - Received prompt ACT | 1/23 | 3.3 | 12/67 | 17.9 |
| - Prescribed 3 days of ACT | 4/23 | 16.8 | 3/67 | 0.7 |
| - Received 3 days ACT | 4/23 | 16.8 | 3/67 | 0.7 |
| - Received quinine | 0/23 | 0 | 13/62 | 35.1 |
| - Received Amodiaquine (not recommended) | 3/23 | 22.3 | 2/62 | 3.8 |
| - Referred with IM quinine or IM, EV or rectal Artesunate | 0/5 | 0 | 2/4 | 4.5 |
| Test negative for malaria | 5/37 | 13.5 | 4/92 | 4.3 |
| - Received ACT | 1/5 | 4.0 | 0/4 | 0 |
| - Received quinine | 0/5 | 0 | 0/4 | 0 |
| - Received Amodiaquine | 0/5 | 0 | 0/4 | 0 |
| - Referred with IM quinine or IM, EV or rectal Artesunate | 0/1 | 0 | 0/0 | 0 |
| Unknown results for malaria test | 9/37 | 19.0 | 21/92 | 24.9 |
| Malaria clinical diagnosis | 21/55 | 23.8 | 123/133 | 93.4 |
| - Received ACT | 1/21 | 0.2 | 13/123 | 7.6 |
| - Received prompt ACT | 0/21 | 0 | 10/123 | 6.4 |
| - Prescribed 3 days of ACT | 1/21 | 0.2 | 4/123 | 1.5 |
| - Received 3 days ACT | 1/21 | 0.2 | 2/123 | 0.1 |
| - Received quinine | 1/20 | 2.4 | 19/123 | 26.3 |
| - Received amodiaquine | 1/20 | 5.0 | 7/113 | 2.2 |
| - Referred with IM quinine or IM or rectal Artesunate | 0/1 | 0 | 0/1 | 0 |
| Diarrhoea received zinc | 7/31***** | 31.3 | 4/86 | 5.5 |
| Diarrhoea received zinc for 14 days | 0/31 | 0 | 0/86 | 0 |
| Diarrhoea received ORS | 6/31 | 22.1 | 8/86 | 5.6 |
| Diarrhoea received ORS and zinc | 1/30 | 0.3 | 0/86 | 0 |
| Suspected pneumonia received amoxicillin or cotrimoxazole | 1/9 | 18.7 | 4/15 | 33.0 |
| Suspected pneumonia received 5 days of amoxicillin or cotrimoxazol | 1/9 | 18.7 | 4/15 | 33.0 |
| Suspected pneumonia received any antibiotic****** | 2/9 | 20.0 | 5/15 | 50.3 |
| Cough received amoxicilline or cotrimoxazol | 7/34 | 30.1 | 30/104 | 27.3 |
| *Weighted estimates. **A high number of carers were not told about the number of days to give the medicines. ***Was prescribe and took ACT for 7 days. **** Only in the Volta Region as CBAs are only provided with malaria treatment. ***** Zinc was taken for 10 days; ******amoxicillin, PNC V, ampicillin, cotrimoxazole, cefuroxime, Azitromicin, cloxacilline, eritromicin flucloxacilline, or chloramphenicol. | | | | |
