# Supplementary material for: Integrated community case management and community-based health planning and services: a cross sectional study on the effectiveness of the national implementation for the treatment of malaria, diarrhoea and pneumonia
Source: Malar J. 2016 Jul 2;15:340. doi: 10.1186/s12936-016-1380-9 (PMC4930600; doi:10.1186/s12936-016-1380-9)
Supplement: Supplementary file 4 — 10.1186/s12936-016-1380-9 Places and reasons for seeking care elsewhere after visiting CBA or a CHPS by region. [file 12936_2016_1380_MOESM4_ESM.docx]

**Additional file 4. Places and reasons for seeking care elsewhere after visiting CBA or a CHPS by region.**

|  | **Volta Region** | | **Northern Region** | |
| --- | --- | --- | --- | --- |
|  | **n/N** | **%*** | **n/N** | **%*** |
| **Seeking care elsewhere after CBA** | **28/90** | **42.4** | **4/8** | **63.3** |
| Seeking care elsewhere in case of fever | 25/28 | 82.6 | 4/4 | 100 |
| Seeking care elsewhere in case of diarrhoea | 12/28 | 66.4 | 3/4 | 36.8 |
| Seeking care elsewhere in case of suspected pneumonia | 6/28 | 10.2 | 1/4 | 20.3 |
| Second provider sought | |  |  |  |
| - CHPS | 4/28 | 54.5 | 2/4 | 68.3 |
| - Health Centre | 7/28 | 21.8 | 1/4 | 11.3 |
| - Hospital | 10/28 | 16.3 | 1/4 | 20.3 |
| - Licensed Chemical seller | 3/27 | 2.2 | 0 | 0 |
| - Private health facility | 4/28 | 4.9 | 0 | 0 |
| Reasons for seeking care elsewhere | |  |  |  |
| - Not getting better | 24/28 | 98.7 | 2/4 | 25.5 |
| - To buy medicines | 3/28 | 1.1 | 2/4 | 74.5 |
| - CBA not available | 1/28 | 0.1 | 0 | 0 |
| **Seeking care elsewhere after CHPS** | **14/61** | **28.0** | **21/228** | **7.9** |
| Second provider sought |  |  |  |  |
| - CHPS | 2/14 | 11.6 | 1/21 | 9.2 |
| - Health Centre | 0/14 | 0 | 9/21 | 23.8 |
| - Hospital | 3/14 | 1.1 | 4/21 | 49.5 |
| - Licensed Chemical seller | 8/14 | 50.4 | 6/21 | 16.8 |
| - Drug peddler | 1/14 | 36.7 | 0/21 | 0 |
| - Traditional healer | 0/14 | 0 | 1/21 | 0.5 |
| Reasons for seeking care elsewhere |  |  |  |  |
| - Not getting better | 5/14 | 51.7 | 20/21 | 99.4 |
| - To buy medicines | 8/14 | 42.4 | 1/21 | 0.5 |
| - Nurse/doctor not available | 1/14 | 5.8 | 0/21 | 0 |

*Weighted estimates
